# Ethanol Production from Extruded Thermoplastic Maize Meal by High Gravity Fermentation with *Zymomonas mobilis*

**DOI:** 10.1155/2014/654853

**Published:** 2014-11-03

**Authors:** Mayeli Peralta-Contreras, Edna Aguilar-Zamarripa, Esther Pérez-Carrillo, Erandi Escamilla-García, Sergio Othon Serna-Saldívar

**Affiliations:** ^1^Centro de Biotecnología FEMSA, Tecnológico de Monterrey, Avenida Eugenio Garza Sada 2501 Sur, Col. Tecnológico, 64849 Monterrey, NL, Mexico; ^2^Centro de Investigación y Desarrollo en Ciencias de la Salud/UOIE, Universidad Autónoma de Nuevo León (UANL), Avenida Carlos Canseco s/n con Avenida Gonzalitos, Mitras Centro, 64460 Monterrey, NL, Mexico

## Abstract

A comparative study of extruded and ground maize meals as raw materials for the production of regular (12°P) and high gravity (20°P) worts was devised. Extruded water solubility index (WSI) was higher (9.8 percentage units) and crude fat was lower (2.64 percentage units) compared to ground maize. Free-amino nitrogen compounds (FAN), pH, and glucose were evaluated in regular and high gravity worts produced from ground or extruded maize. Extrusion improved glucose content and ethanol yield. In 20°P mashes, extrusion is enhanced by 2.14% initial glucose compared with regular ground mashes. The 12°P and 20°P extruded treatments averaged 12.2% and 8.4% higher ethanol, respectively, compared to the uncooked counterpart. The 20°P worts fermented with *Zymomonas mobilis* produced 9.56% more ethanol than the 12°P counterpart. The results show that the combination of extrusion and fermentation of 20°P worts improved ethanol yield per kg flour until 20.93%. This pretreatment stimulates *Z. mobilis* fermentation efficiency.

## 1. Introduction

Bioethanol is produced from the fermentation of sugars obtained from biomass. Bioethanol feedstock can contain either sucrose (e.g., sugarcane, sugar beet) or starch (e.g., corn, wheat) or be a lignocellulosic material (e.g., sugarcane bagasse, wood, and straw). Corn and sugarcane are the feedstock used in the US and in Brazil, respectively, which are the largest ethanol producers in the world [1]. Although maize grain has the highest yield for ethanol conversion (360 liters of ethanol per metric ton) and it is considered the majority source of the fuel ethanol industry in the United States, technological innovations are required in order to provide faster and highest conversion yields of carbohydrates to ethanol [2–4].

There is growing interest to develop several new technologies, especially through mechanical, chemical, and biological processes to optimize ethanol produced from maize [5, 6]. An interesting approach is the implementation of thermoplastic extrusion, because it modifies starch to dextrose and lowers energy needs for high pressure steam. This continuous cooking process pregelatinizes the starch using a combination of moisture, pressure, heat, and mechanical stress [7–9]. Even though the temperature required to complete starch gelatinization is between 80°C and 100°C, the extruder screw speed causes an important pressure increase and the rupture of the food matrix. Extrusion technology allows quick gelatinization using less processing water and short residence time compared to the traditional liquefaction process which could reduce energy and costs. Maize extrudates can be fully and efficiently hydrolyzed in order to elevate the bioavailability of potential sugars [10, 11]. Therefore, the physical and chemical characteristics after the extrusion process have been an important research topic. Carvalho et al. observed on extruded cereal grits changes in particle size distribution which modified the characteristics of the resulting expanded products [11]. On the other hand, Naidu et al. mentioned that the particle size distribution from uncooked cereal meals may affect the starch digestion which could impact the enzymatic hydrolysis and changes during saccharification and fermentation [12].

Fermentation based on* Saccharomyces cerevisiae* has been widely used for the bioethanol and alcohol beverage industries. However,* S. cerevisiae* presents some disadvantages at higher sugar or ethanol concentrations reducing yeast viability and ethanol yield [13]. The research of ethanol-tolerant microorganisms has been highly relevant for fermentation processes. Among them,* Z. mobilis *could produce a 97% theoretical yield of ethanol to glucose, while* S. cerevisiae* only can achieve 90–93% [13–15].

*Z. mobilis* has positioned as an ethalogenic organism with potential industrial application because it possesses a metabolism that yields a molecule of ATP per glucose molecule, and produces less biomass. Also, these strengths include the conversion of 95% of glucose into ethanol (with a minor percentage of 3% of incorporation into its cellular mass), elevated growth rates, tolerance to acidic conditions, no requirement for oxygenated conditions, cost reduction in the aeration, elevated osmotolerance in a substrate/product ratio (because the hopanoids act as stabilizers to the cellular membrane), and reduced nutritional requirements [16]. Moreau et al. have studied ethanol tolerance exhibited by this bacterium based on the hopanoids and other membrane lipids [17].

In characterization studies, the initial sugar concentration is an important parameter for* Z. mobilis* growth; however, high glucose concentrations could induce inhibition ethanol production [18]. High gravity (HG) fermentation technology is used for industrial scale production of bioethanol. HG technology is defined as “the preparation and fermentation to completion of mashes containing 20 g dissolved solids per 100 g mash.” Its benefits include a decrease in process water requirements and energy costs and increased productivity and ethanol concentration in the products without extra capital expenditure [13]. On the other hand,* S. cerevisiae *suffers stress by environmental factors, such as nitrogen limitation, which induces a decline in fermentation activity noted during the early stages of fermentations and can cause sluggish or stuck fermentations [17].

The aim of the current work was to study the effect of thermoplastic extrusion and to propose high gravity ethanol fermentation with* Z. mobilis *ATCC 31823, comparing 12°P and 20°P worts. Despite the importance of the high gravity worts, no previous reports have explored the effects of extruded and ground corn meals with this particular fermentation bacterium.

The pretreatment technology (dry milling and extrusion) and elevated concentration of solids (12°P or 20°P) were compared by the fermentation samples during 72 h. Also, the pH, °Plato, glucose consumption, free-amino nitrogen compounds (FAN), and ethanol concentrations were determined in the present study.

## 2. Materials and Methods

### 2.1. Grains

Commercial regular yellow maize was purchased in a local market and cleaned with air aspiration (Seedburo Equipment Company) to remove foreign material or dockage. Then, the grains were milled with a 2 mm screen in a Wiley mill (Arthur Thomas, Philadelphia, PA, USA) to obtain the sample ground meal as the methodology applied by Chuck-Hernandez et al. [19].

### 2.2. Extrusion Process

The resulting maize meal was separated into two batches: one was stored and the other reserved for the extrusion trials. A twin extruder (BCTM-30 Buhler, Switzerland) with an *L*/*D* = 20 was used to process the maize meal. The extruder operated at 250 rpm and the last section of the extruder barrel was set at 120°C. The screw configuration was selected to induce high levels of shear towards the end of the screws to induce starch gelatinization. Subsequently, the extrudates were collected and samples were placed into a dessicator. Then, extrudates were dried at 60°C in a convection oven and milled; the minimal drying was to control the final moisture before storage. Finally the chemical compositions of regular and extruded maize flours were determined.

### 2.3. Chemical Analyses

The chemical composition of the raw and extruded materials was determined by the following parameters: moisture, crude protein, crude fat, crude fiber, and ash. These assays were performed according to approved methods 44-15 A, 46-13, 30-20, 32-10, respectively [19]. In addition, the total starch content was measured according approved method 76-17 with a commercial kit of Megazyme [19]. Finally, the phenolic compounds assay was performed with the Folin reagent, subsequent to the addition of carbonate solution to the methanolic extracts [19].

### 2.4. Physical Analysis

The water absorption (WAI) and the water solubility (WSI) indexes were determined according to Anderson et al. [20]. Briefly, the ground and extruded meals were suspended in water at room temperature for 30 minutes, gently stirred, and then centrifuged at 3000 ×g for 15 minutes. The supernatant was decanted into an evaporating dish. The WSI was expressed as the weight of dry solids in the supernatant, whereas the WAI was the weight of gel obtained after removal of the supernatant.

In addition, a particle size test was determined in a Rotap (Duratap Model DT168, Advantech Mfg., New Berlín, WI, USA). To perform the distribution size analysis, the maize or extruded flours were placed for 10 min in a Rotap equipped with a nest of coarse (mesh size number 30 and number 40), medium (mesh size number 60 and number 80), and fine (mesh number 100) sieves and a bottom collection pan [5].

### 2.5. Microorganisms and Preservation

The* inoculum* was prepared with the bacterium* Z. mobilis* ZM481 (ATCC 31823) grown in a culture medium referenced as RM ATCC 1341 (containing per liter of water adjusted to pH 6 yeast extract, 10 g; glucose, 20 g; and KH_2_PO_4_, 2.0 g). The media and glucose were sterilized separately by autoclaving at 121°C for 15 min. Finally, the glucose was mixed with the rest of the media components in sterile conditions (Labconco Model 36212, Kansas City, MI, USA).

The culture was maintained without mixing at 30°C in an incubator (VWR International, Model RF1575). The adequate phase growth to harvest the cells was considered in the 75% of the exponential phase with a count of 1.2 × 10^7^ cells/mL and the inoculum aliquots were spectrophotometrically monitored at 600 nm [5]. Following this growth condition, a volume between 7.5 and 10% v/v of the culture was inoculated to the batch fermentations [19]. The microorganism shows an accelerated growth between 10 h and 15 h; this period of time was considered to collect the inoculum and to carry out the fermentation experiments.

### 2.6. Fermentation Process

#### 2.6.1. Cereal Mashes Preparation

The raw and extruded maize meals were liquefied with thermoresistant *α*-amylase (Liquozyme SC DS, Novozymes, Bagsvaerd, Denmark) according to previous investigations [5, 18]. Then, hydrolyzates were saccharified with amyloglucosidase (Dextrozyme, Novozymes, Bagsvaerd, Denmark) in preparation for fermentation trials. The saccharification was performed at 60°C during 17 h in an incubator shaker (VWR International, Model RF1575) and the worts were diluted with distilled water to 12 or 20°P concentrations [21]. The gravity (expressed as °P) was measured using a digital refractometer (CATAGO HRS 500, Bellevue, WA, USA).

#### 2.6.2. Fermentation System

After the °Plato (°P, gravity) concentration was adjusted, the worts were pasteurized in a water bath and transferred to sterilized Erlenmeyer flasks without additional supplementation. Finally, worts were inoculated in sterile conditions (Labconco Model 36212, Kansas City, MI, USA) and incubated at 30°C without mixing for 72 h fermentation [21].

### 2.7. Analytical Methods

The pH, °P, free-amino nitrogen (FAN) compounds, glucose, and ethanol were measured during fermentation. A potentiometer was used (Orion, Model 1230, Germany) for pH determinations in cereal mashes samples. In addition, the °P was measured directly from fermenting mashes placing a 50 *μ*L aliquot in a digital refractometer (CATAGO HRS 500, Bellevue, WA, USA). Both parameters were measured after the sample was collected from the fermentation system.

The collected samples were directly preserved at −20°C for further colorimetric and chromatographic analyses. The amount of FAN generated during fermentation was determined by reaction with ninhydrin according to the Official Method 945.30 [19]. The glucose content was determined by HPLC-RI (Waters 2414, Milford, MA, USA) equipped with an ion-exchange column (Aminex HPX-87H, Biorad Hercules, CA, USA) as described previously by Chuck-Hernandez et al. [19]. Ethanol concentration was measured with Gas Chromatography-FID equipped with a HP-Innowax (30 m × 0.53 mm × 1 *μ*m) column [21].

### 2.8. Experimental Design and Statistical Analysis

The research was conducted in two-level factorial design designed to evaluate two effects: type of feedstock (extruded or ground maize meals) and the concentration of wort (12 or 20°P). The experiments were performed by triplicate. Statistical analysis was performed on the final pH, °Plato, and FAN concentration using the software Minitab 16 (Minitab Inc., State College, PA). The chemical and physical properties were analyzed with descriptive statistics using means and standard deviations.

## 3. Results and Discussions

### 3.1. Chemical Composition

The chemical composition of feedstocks is presented in Table 1. Ground maize showed similar moisture, ash, crude fat, and protein content to the reported composition for corn meals in the literature [22]. The average amounts of protein, ash, and moisture for the extruded meal had similar values compared to its ground counterpart; however, a significant reduction of 36.66% of the phenolic compounds and 86.58% of the crude fat was found in the extruded treatment (Table 1). Wang and Ryu studied the changes induced by extrusion in corn phenolic compounds; they reported a decrease in total phenolics from 59.12 to 21.82 mg GAE/100 g of dry basis when extrusion at 101.5°C was applied to raw material, because phenolic compounds are less resistant to heat and may alter natural properties [23] which present similarities to the results found in Table 1. Chuck-Hernandez et al. found that phenolics compounds did not affect ethanol production in cereal mashes [19]. Also, Carvalho et al. reported protein ranging from 6.15 to 8.04 g/100 g and for crude fat from 0.57 to 0.79/100 g in extruded corn meals [11]. The protein (8.09 g/100 g) content was close to the high-level value for the extruded meal whereas the crude fat (0.42 g/100 g) was within reported values. Ethanol improvement could be partially explained in case of extruded material because of the physicochemical changes and the increase of the superficial area. Hernot et al. found a decrease in the organic matter concentrations after extrusion of native corn substrates, which suggests that high temperatures could breakdown thermolabile structures in crude fat [24]. The moisture was carefully measured; samples were collected in a desiccator after thermoplastic extrusion to avoid moisture loss for evaporation when the extruded samples reached room temperature and the moisture assay was performed.

Total starch values indicate a slight decrease after extrusion cooking and had a variation smaller than 9% when the raw and processed corn meals were compared, which suggests a similar trend to previous studies [25]. Changes in chemical properties suggest that during extrusion cooking the properties of maize meals could be modified because of polymer starch disintegration at 120°C [9].

### 3.2. Physical Properties

The experimental results shown in Table 1 suggest that thermoplastic extrusion changed the distribution of particulates, water solubility (WSI), and water adsorption (WAI) indexes. The process increased the solubility of the extruded corn meal and enhanced the yield of finer particles after milling (Table 1). WAI measures the amount of water absorbed by starch and can be used as an index of gelatinization, whereas the WSI parameter is often used as an indicator of degradation of molecular components, measuring the amount of soluble components released from the starch after extrusion [7]. Both parameters reported higher experimental values for the extruded maize flour compared to the ground meals, which could be related to the degradation of starch and gelatinization, because of the high temperature during extrusion. Also, the dipolymers in the raw materials were subjected to protein denaturalization, starch glue formation, and plasticization of the complete volume [26]. In addition, the pressure change at the end of the die caused the starch to plasticize, which in conjunction with temperatures above 100°C produced water evaporation and expansion with the creation of a foam-like expanded product. In the extrusion process, the distribution of particle size was found to be a higher fraction oriented to the medium (149–420 nm) and small sizes (<149 nm), in comparison to the ground meals. In the case of the ground maize, the particle size distribution favored coarser fractions (Table 1). The particle size affects speed of enzyme hydrolysis and is also related to the bioavailability of sugars that the bacterium is able to use for its growth. During extrusion, the maize meal experienced thermomechanic stress conditions modifying its starch structure [6] which are known to impact the wort during fermentation.

### 3.3. The pH Profile of Maize Mashes during Fermentation

**Figure 1:**
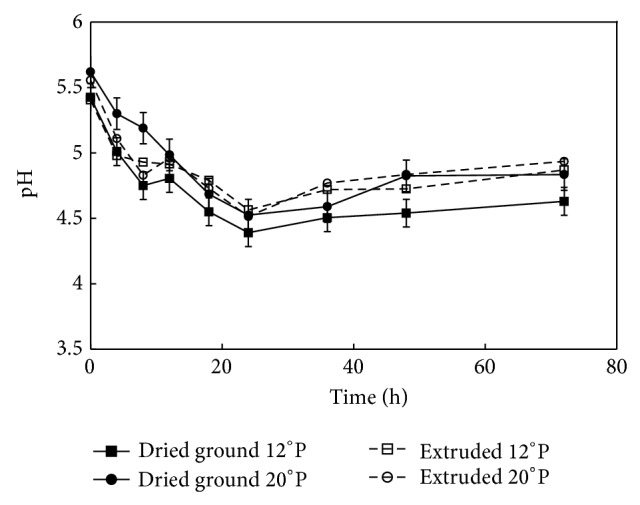
pH profile during fermentation of ground maize at 12 and 20°P and extruded maize at 12 and 20°P mashes by* Zymomonas mobilis* (ATCC 31822).

In fermentation, pH is an important factor because it affects the behavior of bacterium physiology, and a change in this parameter could decrease ethanol yield under certain circumstances [16, 17]. The pH of mashes was 4.75 (±0.15) during the 72 hours of* Z*.* mobilis* fermentation for all the treatments; this general trend can be observed in Figure 1. During fermentation, this bacterium can reduce the pH to 4.0–5.5, but if a pH further drops near 3.5, this indicates that a metabolic disorder may be related to contamination because of high accumulation of lactic and other organic acids [18]. In all of the cases, a maximum decrease of the pH of mashes was between the 10 and 25 h of fermentation, indicating the end of the process of conversion to ethanol [17].

An ANOVA was conducted to evaluate the type of feedstock (dried ground or extruded) and wort concentration (12°P or 20°P) in the final pH in maize mashes after fermentation. This analysis shows that the type of feedstock had a significant influence in the final pH (*P* value < 0.05); however, the concentration was not found to have significant effect. The process type used for the feedstock over the pH could be related to the particle size of the corn meals, and the starch changes might affect the soluble sugars present in the wort during fermentation.

### 3.4. °Plato Profile of Mashes during Fermentation

**Figure 2:**
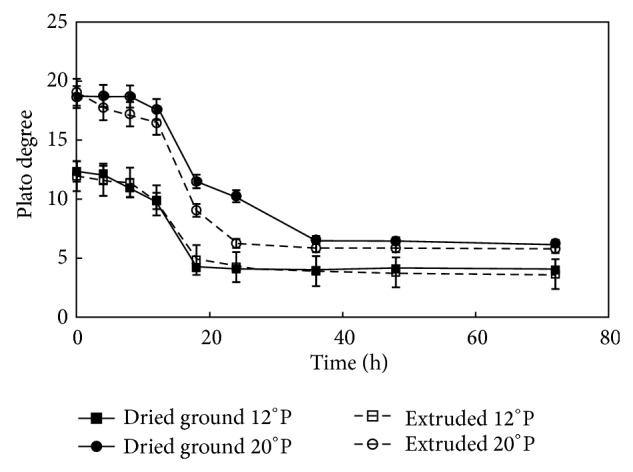
Plato degree profile during fermentation of ground maize at 12 and 20°P and extruded maize at 12 and 20°P mashes by* Zymomonas mobilis* (ATCC 31822).

The °P profile of* Z. mobilis* is an indirect measurement and is shown in Figure 2. °Plato monitoring is a preliminary indicator related to sugar content and can give valuable information about the performance of the microorganism during bioethanol production. Therefore, the Brix or Plato degrees are a reliable measurement for sugar, because of the ability of carbohydrates to affect the refraction index in the solution [27].

In the case of sweet sorghum, the available sugar percentage is significantly associated with Brix, reducing sugar content and pH. Also, a linear correlation was found between °P and total sugar content with sweet sorghum in some studies [28]. In Figure 2, the maize worts showed decreased Brix content during* Z. mobilis* fermentation.

A decrease between 10 and 25 h was observed in the fermentation treatments, which can be correlated with the pH decrease reported in the literature. Fermentation of starchy materials has been reported with ethanol production starting at 24 h [22]; the °P profile can help to estimate an approximate final value for the exponential stage after 25 h of fermentation with the preliminary information.

The type of process and initial concentration affected the final °P value. ANOVA analysis was applied to experimental data and the interaction between factors was found to be statistically significant. This result may suggest that extrusion as a process had an effect on the bioavailability and content of sugar in the maize meals.

Since the 20°P worts contained higher sugar concentration than the 12°P worts, a substrate inhibition by glucose could be present and this phenomenon could imply that concentrated worts might reduce process efficiency leaving residual sugar at the end of fermentation. The 12°P treatments had a higher sugar consumption leaving less residual sugar compared to the 20°P counterparts. A comparison of maize with other starchy substrates, such as sorghum and decorticated sorghum, has been reported extensively in the literature [19, 20].

### 3.5. FAN Consumption of* Z. mobilis* during Fermentation

In yeast fermentation, there are two important components that impact the overall process and efficiency: the initial sugars and the diversity of nitrogen compounds [14]. The nitrogen consumption of* Z*.* mobilis* is comparatively less relevant because its demand is lower compared to* Saccharomyces cerevisiae *[12]. For* S. cerevisiae, *a FAN content of 150 mg/L wort is considered as minimum to achieve good fermentation [19, 21, 22].

The initial FAN content was lower in the evaluated worts, and this reduced amount of nutrients could modify the bacteria metabolism. Even though most* Zymomonas* strains are known to be autotrophic organisms, there are several reports in the literature of defined and minimum nutrients for* Z. mobilis* to achieve high performance fermentation [16]. Relatively little is understood concerning the specific influence on growth and assimilation of individual components of this bacterium, which requires lower nutrients in the media culture with respect to yeast in order to support its growth during fermentation [28].

**Figure 3:**
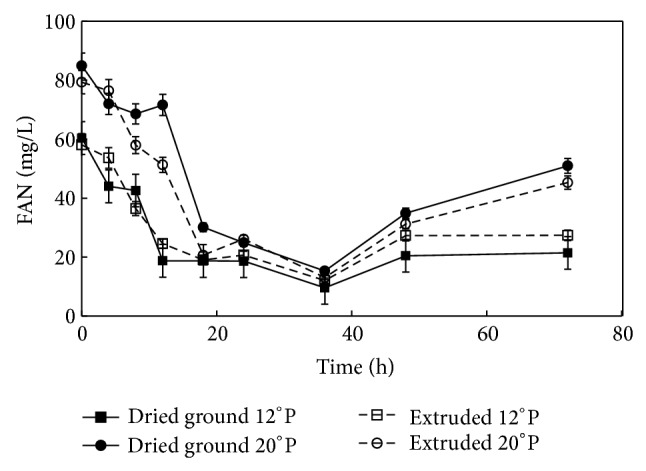
FAN (mg/L) concentration during fermentation of ground maize at 12 and 20°P and extruded maize at 12 and 20°P mashes by* Zymomonas mobilis* (ATCC 31822).

**Figure 4:**
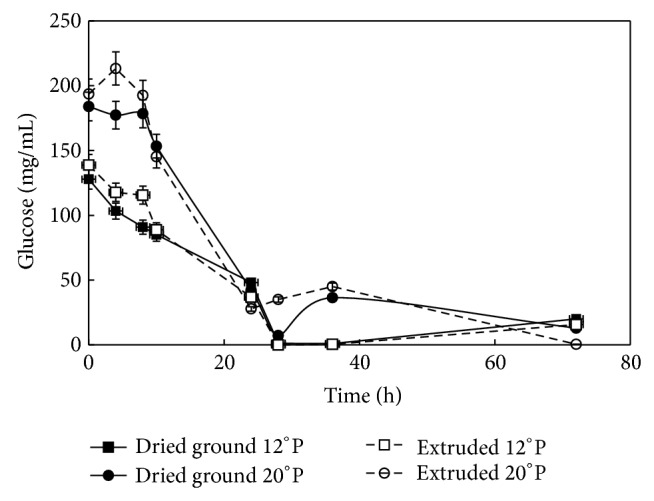
Glucose (mg/mL) consumption during fermentation of ground maize at 12 and 20°P and extruded maize at 12 and 20°P mashes Z*ymomonas mobilis* (ATCC 31822).

In Figure 3, FAN consumption presents a similar trend in the four treatments. The major decrease in FAN compounds was observed between 8 h and 18 h, which was found to have similarity with the time when the bacteria started higher glucose consumption (Figure 4). Whereas FAN concentration decreased 15.5% during the first 10 h fermentation in the 20°P mashes, 12°P mashes remained similar (*P* > 0.05). The total FAN consumption in the worts presented a lower value at 38 h in all treatments, and this behavior could be related to the death phase of the bacterial population. The increase of amino acids after the 40 h of process could be attributed to the autolysis of* Z*.* mobilis* and the hydrolysis of the initial nitrogen compounds into smaller peptides, which increased the FAN content during fermentation [28].

Process type did not affect the initial and final FAN contents in the corn meals, even though the FAN amount was not the optimum reported for other microorganisms; however for the bacterium the conditions were sufficient to fulfill its metabolism [6, 14]. The demand of* Z*.* mobilis* for nitrogen compounds would be necessary to determine in future research.

### 3.6. Glucose Consumption in Maize Mashes during Fermentation

The comparison of the glucose profile as affected by the different treatments is shown in Figure 4. The 20°P worts had a faster consumption of glucose compared to the 12°P counterparts; these experiments were performed in a batch system, to allow studying glucose generation and consumption after saccharification and fermentation, respectively. In the presence of glucose or fructose,* Z. mobilis* is able to grow, but glucose is preferred over fructose because of the specific kinetics of sugar uptake [14]. Figure 4 clearly depicts a decrease of glucose in the first 24 h of fermentation, which is similar to the 25 h period that Rogers et al. reported for a batch culture [16]. The rapid glucose transport rates between external and internal sugar concentrations may contribute to the osmotic adjustment [6, 14]. In terms of the extrusion process, the 20°P extruded maize presented a steeper slope during the first 20 h of fermentation consumption compared to the 20°P ground raw maize. Also, the higher WSI observed in the extruded materials positively impacted glucose consumption between the 4 h and the 20 h due to their higher solubility.

Lawford reported 95% glucose consumption with a process based on corn starch hydrolyzed to contain 162–172 g glucose/L [29]. In the present study, the fermentation experiments were performed without mixing in order to avoid aeration. The aeration on growth and metabolism at high glucose concentration can yield inhibitors such as acetaldehyde. The isozyme alcohol dehydrogenase catalyzes in the microorganism the reduction of acetaldehyde to ethanol; however when fructose is not efficiently phosphorylated by fructokinase, both enzymes depend on the sugar in the culture. Therefore, the microorganism is less efficient on the ethanol batch fermentation [30].

### 3.7. Ethanol Production during Fermentation

**Figure 5:**
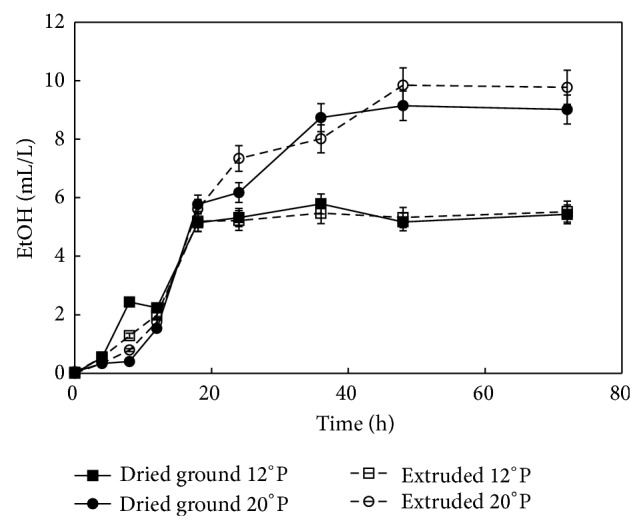
Ethanol (mL/L) production during fermentation of ground maize at 12 and 20°P and extruded maize at 12 and 20°P mashes by* Zymomonas mobilis* (ATCC 31822).

Figure 5 clearly shows that independently of the type of treatment approximately 60% of the ethanol was produced during the first 20 h fermentation. This behavior could be the initial lag phase of the ethanol formation, which corresponded to the transformation phase of the bacterium from the lag to the exponential growth. A further increase of ethanol production occurred during the 20–30 h of fermentation similar to data previously reported by Perez-Carrillo et al. [6]. Results indicated that the proposed thermoplastic extrusion generated a feedstock more adequate for starch conversion and subsequent fermentation compared to the raw meal. The 12°P and 20°P mashes produced from the extruded feedstock yielded 12.2% and 8.4% more ethanol compared to the uncooked counterparts. Ethanol production was higher in the fermentation systems with 20°P compared to worts with 12°P. The high gravity regular and extruded worts yielded 10.8% and 6.1% more ethanol compared to the 12°P counterparts (Table 2) which allows less water consumption during the process. The combination of extrusion mashes show improvements in* Z. mobilis* fermentation until 20.93% compared to the uncooked mashes; the comparative chart is presented in Table 2. In previous work [6] reported ethanol yield at 20°P of 0.27 L/ground grain kg, similar to value obtained in this work at 13°P with uncooked mashes. This study provides a combination that opens a possibility of reducing water consumption and better use of existing fermentation reactors.

## 4. Conclusions

Thermoplastic extrusion improved glucose consumption and bioavailability throughout fermentation especially for high gravity worts. This effect can be related to the finer particle size, solubility, and chemical changes generated during extrusion of maize meals especially in terms of starch gelatinization. Extruded treatments enhanced ethanol concentration by 10.19% compared to ground treatments which positively impact fermentation.* Z. mobilis* showed synergism with raw and extruded 20°P mashes yielding 10.8% and 6.1% more ethanol compared to the 12°P counterparts. This research demonstrates that the combination of extrusion cooking and fermentation of 20°P mashes is advantageous from the ethanol and efficiency viewpoints.

## Figures and Tables

**Table 1:** Physical and chemical properties of processed maize flour used for the fermentation worts.

Parameters (%)	Dried ground	Extruded 120°C
Chemical composition		
Moisture	12.62 ± 0.10	12.90 ± 0.10
Ash content	1.18 ± 0.05	1.18 ± 0.02
Protein	8.84 ± 0.50	8.09 ± 0.18
Crude fat	3.13 ± 0.26	0.42 ± 0.08
Total starch	74.50 ± 0.60	71.77 ± 1.32
Crude fiber	1.24 ± 0.16	2.67 ± 0.12
Phenolic compound^a^	376.39 ± 68.18	238.37 ± 25.19
Physical properties		
WAI	2.82 ± 0.19	4.42 ± 1.26
WSI	7.65 ± 0.27	17.54 ± 6.68
Particle size fraction^b^		
Coarse (>420 mm)	61.68	37.74
Medium (149–420 mm)	35.91	47.66
Fine (<149 mm)	1.65	14.58

^a^Expressed in *μ*g of ac gallic equivalents/dried gram.

^b^Average values. Determined after grinding extruded and dried corn meals in a Wiley mill equipped with a 2 mm screen.

**Table 2:** Ethanol yield during fermentation of yellow maize with or without extrusion treatment at 20 or 12°P wort with *Zymomonas mobilis *(ATCC 31822).

Treatment	°P	L (EtOH)/flour Kg	L (EtOH)/starch Kg
Ground maize	12	0.272 ± 0.016	0.387 ± 0.027
	20	0.305 ± 0.011	0.432 ± 0.011

Extruded maize	12	0.312 ± 0.020	0.437 ± 0.042
	20	0.330 ± 0.100	0.468 ± 0.014
